# Multi-Residency Implementation of an Online Movement Disorders Curriculum Based on Real Patient Videos

**DOI:** 10.5334/tohm.654

**Published:** 2021-09-22

**Authors:** Sara M. Schaefer, Zachary London, Joseph M. Ferrara, Matthew A. McCoyd, Carolyn Cronin, Emily Poole Pharr, Raymond Price, Heather B. Rigby, Scott Vota, Molly Cincotta, Martin D. Slade, Jeremy J. Moeller

**Affiliations:** 1 Yale University School of Medicine, 333 Cedar St. New Haven, CT 06510, US; 2 University of Michigan Medical School, 1301 Catherine St, Ann Arbor, MI 48109, US; 3 Carilion Clinic, 3 Riverside Circle, Roanoke, VA 24016 (current: Prisma Health, Columbia, SC), US; 4 Loyola University Chicago Stritch School of Medicine, 2160 S 1st Ave, Maywood, IL 60153, US; 5 University of Maryland School of Medicine, 655 W Baltimore St S, Baltimore, MD 21201, US; 6 Wake Forest School of Medicine, 475 Vine St, Winston-Salem, NC 27101, US; 7 Perelman School of Medicine- University of Pennsylvania, 3400 Civic Center Blvd, Philadelphia, PA 19104, US; 8 Dalhousie University Faculty of Medicine, 5849 University Ave, Halifax, NS B3H 4R2, CA; 9 Virginia Commonwealth University School of Medicine, 1201 E Marshall St #4-100, Richmond, VA 23298 (current: Bon Secours Mercy Health, 8260 Atlee Rd, Mechanicsville, VA 23116), US

**Keywords:** Graduate medical education, resident education, movement disorders, online learning, flipped curriculum, computer-based teaching modules

## Abstract

**Background:**

In-person didactic education in residency has numerous challenges including inconsistent availability of faculty and residents, limited engagement potential, and non-congruity with clinical exposure.

**Methods:**

An online curriculum in movement disorders was implemented across nine neurology residency programs (six intervention, three control), with the objective to determine feasibility, acceptability, and knowledge growth from the curriculum. Residents in the intervention group completed ten modules and a survey. All groups completed pre-, immediate post-, and delayed post-tests.

**Results:**

Eighty-six of 138 eligible housestaff (62.3%) in the intervention group completed some modules and 74 completed at least half of modules. Seventy-four, 49, and 30 residents completed the pre-, immediate post-, and delayed post-tests respectively. Twenty-five of 42 eligible control residents (59.5%) completed at least one test. Mean pre-test scores were not significantly different between groups (6.33 vs. 6.92, p = 0.18); the intervention group had significantly higher scores on immediate post- (8.00 vs. 6.79, p = 0.001) and delayed post-tests (7.92 vs. 6.92, p = 0.01). Residents liked having a framework for movement disorders, appreciated the interactivity, and wanted more modules. Residents completed the curriculum over variable periods of time (1–174 days), and at different times of day.

**Discussion:**

This curriculum was feasible to implement across multiple residency programs. Intervention group residents showed sustained knowledge benefit after participating, and residents took advantage of its flexibility in their patterns of module completion. Similar curricula may help to standardize certain types of clinical learning and exposure across residency programs.

**Highlights:**

Interactive online tools for resident didactic learning are valuable to residents. Residents learn from interactive online curricula, find the format engaging, and take advantage of the flexibility of online educational tools. Beginner learners appreciate algorithms that help them to approach a new topic.

## 1 Introduction

It is challenging to provide a comprehensive outpatient educational experience to residents due to many factors, among them inpatient clinical demands, a busy outpatient clinical environment, and faculty availability and expertise [1,2,3]. Programs may supplement inconsistent outpatient experiences with didactic activities that have classically occurred in person, but clinical obligations may prevent learners from attending face-to-face sessions, and recently, the COVID-19 pandemic has compounded the need to replace in-person didactics with distance-learning options [4]. Adaptations are variable across programs and include dedicated didactic days or half-days and elaborate cross-coverage arrangements to allow full resident participation in didactics. Furthermore, traditional didactics often suffer from poor temporal correlation with clinical experiences, given that they are offered at a specific date and time to groups of residents, a structure which stymies the timely application of new knowledge. For example, residents may learn about dystonia in a didactic experience while busy on a stroke rotation, and by the time they are in a movement disorders clinic some months later the information is no longer fresh in their minds and ready for application.

Many modern learners have expressed a preference for more interactive and self-directed educational formats [5]. Didactic sessions are traditionally taught in a lecture format, which offers limited opportunities for interaction or application of skills and occurs at the pace of the group rather than the individual learner. Online resources for movement disorders education do exist, most notably through the MDS Education Roadmap offered by the International Parkinson and Movement Disorders Society [6]. This tool splits educational materials into beginner, intermediate, and advanced resources ranging from 20-120 minutes in completion time and is available to all trainees with a free MDS membership. It is an invaluable resource that encompasses a large range of important topics in movement disorders. Many of the sessions consist of traditional hour-long recorded lectures with slides, which are disadvantaged by their lack of interactivity and their length [7]. Studies have shown that medical learner attention spans may be as short as 15 or even six minutes [8,9], and busy neurology residents prefer “bite-sized” learning resources [10]. Some of the newer additions to the MDS Education Roadmap do provide interactive components that likely appeal well to the millennial learner.

One additional barrier to learning movement disorders is the complex language used to describe movement disorders phenomenology. A trainee who is being exposed to movement disorders for the first time is met with a plethora of descriptive terms that are overlapping, occasionally contradictory and inconsistent between resources. For example, chorea may be described as “involuntary, continual, abrupt, rapid, brief, unsustained, irregular movements that flow” [11]. The trainee is tasked with deciding whether abrupt, rapid, and brief are different from one another, how a movement can be unsustained but also continual, and how to compare and contrast one phenomenology from another. While movement disorders experts may perform well without a structured framework underpinning their phenomenological determinations, new learners have been shown to benefit from a structured approach when met with new concepts [10,12].

**Figure 1:**
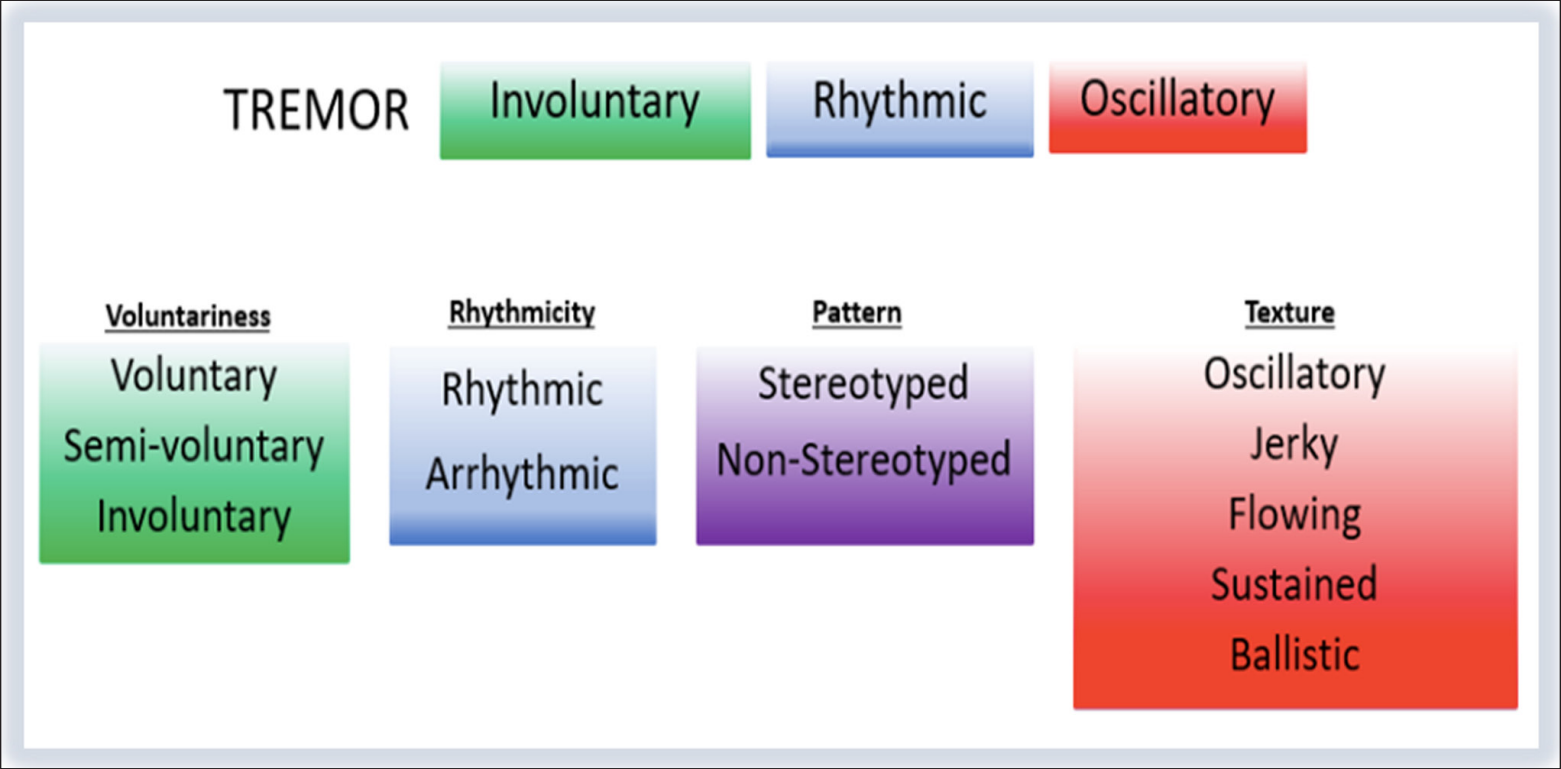
Categories and phenomenological descriptors with example (tremor).

To address the challenges in formal movement disorders education, we studied the implementation of an online, interactive curriculum in movement disorders across several neurology residency programs. The curriculum begins with an introductory video that proposes a structured framework for phenomenological determination, starting with a core set of descriptors and building from the descriptors to the phenomenology in order to inform the diagnosis [10] (***Figure 1***). The remaining curriculum utilizes the framework as a starting point within each module, focusing on individual elements of real patient examinations so that trainees can understand what they should look for in an examination of an undifferentiated movement disorders patient. The curriculum is designed to provide learners who are new to movement disorders the tools to correctly determine phenomenological diagnoses, in the form of short (10–20 minutes), easy-to-digest modules. Video features including voiceover, arrows, slow-motion, freeze frame, and others, alternating with embedded questions and conditional answers, provide a dynamic experience to viewers so that they are able to maximize their learning. The purpose of this study was to assess the impact of this curriculum on knowledge growth, to determine its acceptability among trainees and feasibility of implementation, and to explore patterns of user engagement.

## 2 Methods

### 2.1 Study Setting and Participants

**Figure 2:**
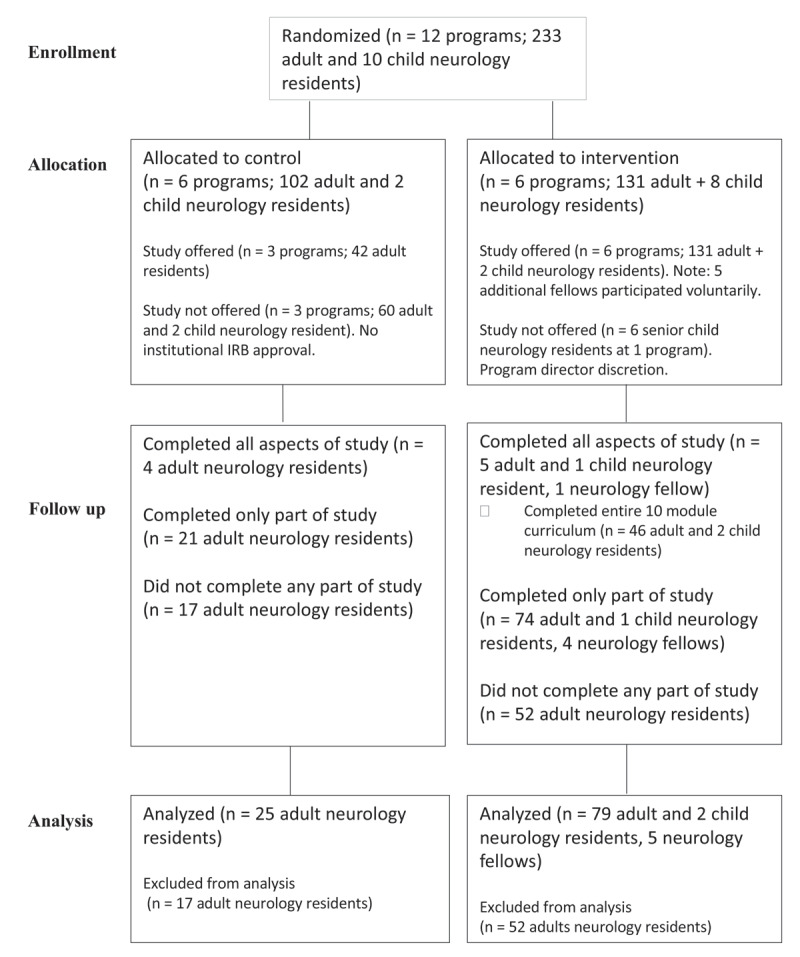
CONSORT statement outlining the enrollment, allocation and follow-up of learners in the trial.

Members of 12 neurology residency programs in North America were recruited on a voluntary basis through an email sent to neurology residency program directors, totaling 233 adult neurology and 10 child neurology residents (***Figure 2***). Data from subjects from the pilot study at the Yale Neurology Residency Program were included with the intervention group; three PGY-5 fellows were part of this cohort [10], and two additional PGY-5 neurology fellows participated in the study from other sites at the discretion of their program directors. All study elements were available through Qualtrics Survey Platform through Yale University, and on a centralized website (*http://movementmodules.yale.edu*).

### 2.2 Study Design

Ten movement disorders modules were designed using online formats to create an interactive, patient video-based curriculum in movement disorders for neurology residents. Further details of the design of this curriculum have previously been published [10]. The 12 original programs were randomized by SMS via random number generation to assign six control and six intervention programs. Institutional Review Board (IRB) approval was sought at each program. After randomization, three programs in the control group dropped out of the study because of unanticipated difficulties obtaining IRB approval. The decision was made to proceed with the study despite these programs dropping out, considering that the alternative would be to wait an entire academic year before implementation. Thus, those three programs were excluded (***Figure 2***). IRBs in general had concerns-- about anonymity of data, program leadership access to the data or to information about individual resident participation, and the implications for coercive influence on residents to participate—that were mostly able to be addressed. The remaining institutions received IRB approval and were included. Residents were consented verbally at their respective programs by program directors or other members of the study team and provided with a consent form at study enrollment and on the study website. All participation was tracked using personal identifiers that were designed to ensure anonymity. Completed study elements were accessible by study members at Yale University through a dual authentication password-protected system.

Intervention group participants were divided within residency programs to assigned pre-, immediate post-, and delayed post-tests in different configurations (e.g., A then B then C, A then C then B, etc.). They were asked to fill out the 10-question pre-test, then complete the modules from June to December 2018. Intervention group program directors were given discretion on methods of module implementation including timing and mandatory vs. optional classification, intended to mimic “real life” implementation of an asynchronous learning resource. Most (four) programs asked residents to complete the modules on their own time over several months. One program used the modules as part of the mandatory orientation curriculum for incoming PGY-2 neurology residents and offered the modules to other residents as an optional resource, and one program offered the modules as part of a dedicated 6-week movement block.

Immediately after module completion, intervention participants were asked to complete the 10-question immediate post-test and survey. In Spring 2019, residents were asked to complete the delayed post-test. Because there was variability in timing of module and test completion, immediate post-test was defined as less than and delayed post-test as greater than 30 days after completion of the final module. The trial was completed at the end of the academic year 2018–2019 due to graduation of participants. Control participants were similarly divided within their programs into pre-, immediate post-, and delayed post-test configurations. Pre-test was defined as completed in 2018, immediate post-test January-March 2019, and delayed post-test April-June 2019, to mirror the intervention group. Control participants did not complete the curriculum or survey.

### 2.3 Outcomes

A pre-test, immediate post-test, and delayed post-test were developed using the principles in the National Board of Medical Examiners guide for item writing [13]. Thirty multiple-choice items were written covering: 1) phenomenology, 2) workup, 3) diagnosis, or 4) treatment. Each item was reviewed by two movement disorders specialists and one neurologist with item-writing training and experience. The items were divided between three 10-item tests (Tests A, B and C). A Likert scale-based survey allowed participants to rate technical and content aspects of the modules and included open-ended questions about participants’ impressions of the modules (Supplemental Materials).

Qualtrics-based user analytics were mined to determine dates and times of module completion. The YouTube analytics function was used to determine audience retention for the introductory video.

### 2.4 Data Analysis

The distributions of the dependent variables utilized in the modeling were relatively normally distributed. As such, linear models (ANOVA, t-test) were deemed appropriate and utilized. T-tests were performed comparing intervention vs. control group scores on pre-, immediate post-, and delayed post-tests. T-tests for pre-and immediate post-tests were performed comparing those in the intervention group who completed ≤ five modules and those who completed 6-10 modules. Delayed post-tests were not included in this analysis since no participants who completed ≤ five modules completed a delayed post-test. Pre-test scores were separately compared using a multivariate model as a function of PGY class and program and using a bivariate model as a function of PGY class alone.

ANOVA calculations were performed modeling the change in test scores as a function of starting score (equals “pre-test” for pre- vs. immediate post-tests and pre- vs. delayed post-tests, and “immediate post-test” for immediate post- vs. delayed post-tests), PGY class, and resident/fellow status. A separate analysis explored the changes only as a function of starting score. The standardized mean difference (Effect size, Cohen’s d) was calculated for the immediate delayed post-tests. Likert-style survey answers were divided into categories relating to 1) technical aspects, 2) quantity/length of modules, 3) enthusiasm about the modules, and 4) perceived gain of knowledge. Scores from 1-5 were assigned to each Likert-style survey answer (poor or strongly disagree = 1, excellent or strongly agree = 5). Within-question as well as categorical means and standard deviations were calculated. Free text responses were single coded and probed for themes by SS.

## 3 Results

### 3.1 Participation

In the intervention group, 133 housestaff were offered participation in the study (131 adult neurology residents, 2 child neurology residents). Five additional neurology fellows participated voluntarily (one movement disorders fellow, four other neurology fellows) for a total of 138 eligible intervention participants. The modules were made mandatory for 57/133 (42.9%) of residents to whom the program was offered. Eighty-six participants (34 PGY2s, 26 PGY3s, 21 PGY4s, and 5 PGY5s) completed at least one module. Two of the PGY-3s were child neurology residents. Most popular subspecialty interests were undecided (n = 22), movement disorders (n = 11), epilepsy (n = 9), and neuromuscular, neuroimmunology, and neurointensive care (each n = 8). Forty-eight participants completed all 10 modules, 20 completed 6–9 modules, and 18 completed five or fewer modules. 10/11 (90.9%) residents who indicated an interest in a career in movement disorders completed at least 6 modules, compared to 79% of the group overall. By PGY level, 79% of PGY2s, 69% of PGY3s, 86% of PGY4s and 100% of PGY5s who started the modules completed 6–10 modules. Of the 86 participants who completed at least one module, 74 completed the pre-test, 49 the immediate post-test, and 30 the delayed post-test. The participating control group included 7 PGY2s, 9 PGY3s, and 9 PGY4s, each completing at least one test, with the most common subspecialty interests being epilepsy (n = 6), stroke (n = 5), undecided, neurointensive care, and movement disorders (each n = 3), and sports medicine (n = 2). Numbers of residents who completed the pre-, immediate post-, and delayed post-tests in the control group were 13, 14, and 13 respectively. Forty-one participants from all six intervention programs completed the modules survey.

The characteristics of each participating residency program are listed in ***Table 1***, as reported by the respective program directors. There was a wide range in mandatory clinical experiences in movement disorders. Many programs offered additional elective or selective experiences. Didactic experiences for residents were similarly variable. In addition to information listed in ***Table 1***, one intervention program had a 6-week educational block in movement every 3 years, which occurred during the study period.

**Table 1:** Baseline characteristics of participating residents and programs (median and ranges).


	CONTROL GROUP	INTERVENTION GROUP

Median (range) number of residents in program (PGY2-PGY4)	18 (6–18)	18.5 (10–35)

Residents with interest in movement disorders	3/25 (12%)	11/86 (13%)

Year of training for participating residents		
– PGY-2 – PGY-3 – PGY-4 – Other	7/25 (28%) 9/25 (36%) 9/25 (36%) 0	32/86 (37%) 26/86 (30%) 21/86 (24%) 5/86 (6%)

Median (range) number of movement disorders faculty members	4 (1–6)	4.5 (2–10)

Median (range) number of mandatory half-day clinical experiences in movement disorders throughout residency	24 (20–70)	7.5 (0–34)

Median (range) number of hours of didactic teaching in movement disorders per year	16 (11–30)	12.5 (5–15)

Median (range) time from completion of pre-test to immediate post-test, in days.*	152 (102–157)	26 (0–143)

Median (range) time from completion of pre-test to delayed post-test, in days*	210 (167–249)	204 (67–399)

Median (range) time from completion of final module to delayed post-test, in days.*	–	173 (38–345)


* For controls, “pre-test” was defined as completed in 2018, “immediate post-test” January–March 2019, and “delayed post-test” April–June 2019, to mirror the intervention group.

### 3.2 Quantitative Results

Results of the adjusted models reveal there was no statistically significant difference in scores between intervention and control subjects on pre-tests (mean scores 6.33 vs. 6.92, p = 0.16) (***Table 2***). Differences between PGY class and program were not statistically significant on pre-tests (p = 0.10 and 0.08 respectively). The intervention group achieved statistically higher scores on the immediate (8.00 vs. 6.79, p = 0.001) and delayed (7.92 vs. 6.92, p = 0.01) post-tests compared to the control group. The overall effect size of the curriculum was 0.88 on the immediate post-test and 0.68 on the delayed post-test, suggesting a medium-sized effect of the intervention. Participants who completed 6–10 modules demonstrated similar pre-test scores compared to those who completed five or fewer modules (6.36 vs. 6.22, p = 0.763), but had significantly better immediate post-test scores (8.26 vs. 6.43, p < 0.001) (***Table 3***).

**Table 2:** Comparison of test scores (out of 10) between control and intervention groups.


	CONTROL GROUP MEAN (STD DEV)	INTERVENTION GROUP MEAN (STD DEV)	P-VALUE	COHEN’S EFFECT SIZE (d)

**Pre-test**	6.92 (1.12)	6.33 (1.50)	0.18	

**Immediate post-test** **(<30 days)**	6.79 (1.35)	8.00 (1.39)	0.001	0.88

**Delayed post-test** **(>30 days)**	6.92 (1.71)	7.92 (1.19)	0.01	0.68


**Table 3:** Comparison of test scores (out of 10) between intervention participants who completed ≤ five modules vs. 6–10 modules.


	TEST	NUMBER OF MODULES COMPLETED	n	MEAN	STD DEV	P-VALUE

**Pre**	t-test	5 or Fewer	13	6.22	1.28	0.763

		6–10	61	6.36	1.56	

**Immediate Post**	t-test	5 or Fewer	7	6.43	1.27	<0.001

		6–10	42	8.26	1.23	


### 3.3 User Analytics

The range of time to curriculum completion for participants who completed more than half of the curriculum (>5 modules) was 1–174 days. For those who completed all 10 modules the median was 16.7 days, including five participants completing them in a single day. The majority of modules (including the introductory video) were completed during daytime hours (7am–5pm; n = 421; 56.3%), followed by the evening (5pm–10pm; n = 227; 30.3%), and overnight (10pm–7am; n = 100; 13.3%). Including all modules, median time for module completion was 14.7 minutes, ranging from a median completion time of 7.6 minutes for the introductory video to 32.9 minutes for Module 5. 74% of introductory video viewers were retained until the last slide, after an initial 20% drop-off in the first 30 seconds of the video. The median time to completion of the introductory video of 7.6 minutes actually exceeded the 6.49-minute running time of the video, indicating that residents likely watched parts repeatedly, and indeed there were small audience retention peaks at slides exploring basic concepts of phenomenology vs. diagnosis, and hypokinetic descriptors.

### 3.4 Survey Results

Means for all survey questions were ≥ 4.0 and means were ≥ 4.5 for 13/18 questions (***Table 4***). The enthusiasm category demonstrated the highest categorical mean of 4.67. 87.8% of respondents “strongly agreed” that they would recommend the modules to other residents. In the qualitative responses, the main themes that emerged were 1) the structure was conducive to learning, 2) the modules were engaging, and 3) the content was high yield. Participants appreciated the “bottom up” framework, feeling that it was “practical” and “accessible.” Many commented that the repetition of key points contributed to learning. The interactivity was often cited as contributing to engagement: “having an interactive and didactic mix definitely kept me engaged.” The brevity and simplicity of each module also contributed to engagement, as did the use of real patient videos with a variety of audiovisual features. Finally, residents found the modules to be an important adjunct to other learning experiences, described by one respondent as an “indispensable tool for my training.” No respondent commented on reasons for not completing all modules. For areas of improvement, most comments were technical (e.g., ease of toggling between pages). Several respondents commented that the modules were repetitive (which was also listed by some as an advantage) and one survey-taker remarked that “they took a long time to complete.” Several survey respondents requested modules of increasing complexity, with additional focus on pathophysiology and treatment.

**Table 4:** Individual question and categorical means of survey questions.


CATEGORY	QUESTION (ABBREVIATED)	MEAN (SD)	CATEGORICAL MEAN (SD)

Technical	Video Image	4.48 (0.75)	4.48 (0.10)

	Video Sound	4.50 (0.68)	

	Ease of access	4.54 (0.71)	

	Questions linked to answers	4.45 (0.64)	

	Table of contents	4.28 (0.78)	

	Text	4.58 (0.59)	

	Embedded images	4.55 (0.60)	

Quantity	Right duration	4.68 (0.76)	4.63 (0.07)

	Right number	4.58 (0.87)	

Enthusiasm	Good use of time	4.70 (0.82)	4.67 (0.10)

	Enjoyment	4.58 (0.84)	

	Recommend for other subspecialties	4.72 (0.79)	

	Recommend for other residents	4.79 (0.73)	

	Appropriate for learning level	4.58 (0.84)	

Knowledge	Understand basic concepts	4.70 (0.82)	4.56 (0.15)

	Understand difficult concepts	4.45 (0.90)	

	“Get” concepts didn’t understand	4.41 (0.94)	

	Confident in diagnosis	4.67 (0.84)	


(SD = standard deviation).

## 4 Discussion

In this extension of our previously-published pilot study [10], we aimed to assess the generalizability of this online curriculum in movement disorders for trainees at a variety of neurology programs. The online modules provided a structured, standardized movement disorders curriculum at programs that were highly variable in terms of availability of local expertise and the range of clinical and didactic movement disorders experiences available to residents. At the core of these modules were videos of actual patients with movement disorders, allowing for standardized, structured virtual exposure to patients with a range of abnormal movements. Our results suggest that the curriculum was acceptable to many residents, feasible to implement in a variety of training environments with different patterns of didactic and clinical schedules and different methods of implementation, and promoted learning as evidenced both objectively on pre- vs. post-test data and subjectively in survey responses. The majority of participants who started the curriculum completed all 10 modules, which suggests that most residents who tested the curriculum found it useful enough to continue to use.

The flexible nature of the curriculum was an advantage that was exploited by both programs and individuals. Programs made variable choices as to the integration of this curriculum into their program schedules. Because it was easily accessible online, residents could fully engage with the curriculum at any time, whether or not there were local faculty available for teaching. In addition, residents could see clear examples of a broad range of movement disorders, which may not always be possible in an unstructured outpatient clinic setting. A study of neurology residents in 2015 reported that most learners have access to a mobile device while in the clinical setting, and so there are minimal technological barriers to this type of web-based curriculum [14]. Participants completed the modules over highly variable periods of time and at all hours of the day and night, which would be impossible to accommodate with in-person didactic education. Computer-based teaching modules (CBTMs) have the advantage of allowing for greater learner convenience, learner-driven pacing, and ability to disseminate across institutions [15], as we have seen in this study. This study did not directly compare traditional didactics to CBTMs, but CBTMs have previously been shown to have improved or comparable teaching efficacy when compared to lectures, small groups, or self-study [15,16].

Participation at the institutional and resident levels was a major limitation of this study, opening the possibility for participation bias. Three control programs did not ultimately achieve IRB approval, or achieved approval too late, which may be partially attributable to differences in IRB application processing times and approval rates between institutions. It is possible that when a program was assigned to the control group, there was less motivation to proceed quickly toward IRB approval. Future multi-center educational studies would benefit from delaying randomization of programs until after all IRBs have approved the study protocol, although in our study, awaiting the additional IRB approvals would have delayed implementation by one year. Because there were only three control sites instead of the anticipated six sites, there was an approximately 30% decrease in statistical power. This could result in an inability to detect a small but significant difference in the pre-test results between the two groups, but in spite of this decreased statistical power, there was a significant difference in immediate post- and delayed post- test results between groups. Additionally, although the intervention and control groups had similar median numbers of residents (18.5 vs. 18 respectively) and movement disorders faculty (4.5 and 4 respectively), the intervention group did have higher numbers of both, which could have skewed the results in favor of the intervention group (***Table 1***). We would anticipate that if these were significant predictors, the pre-test scores would have differed between groups, which they did not. One intervention program also did include the modules as part of their pre-planned dedicated movement disorders block, but it should be noted that on average the intervention group actually had less didactic exposure to movement disorders outside of the modules than the control group over the study period. The fact that the modules were implemented at each program differently simultaneously spoke to the flexible nature of the curriculum, which can be adapted to a variety of programs with different baseline didactic and clinical structures, and also makes cross-comparisons more challenging.

Thirty-eight percent of eligible intervention participants did not complete any modules, which may be related to the voluntary designation of the curriculum at some programs. Programs that made the curriculum mandatory generally had better resident participation, although one program that made the curriculum mandatory still had very poor participation. Another explanation is the lack of accountability given the use of anonymous identifiers to track participation. Programs with better participation checked in with the principal investigators throughout the study period to assess program progress, and sent reminders to their residents based on that progress. Therefore, engagement of local residency leadership and regular communication with residents may have boosted participation. The program that offered the curriculum as part of orientation had very high rates of completion, possibly due to residents getting sufficient time to complete the curriculum without excessive clinical obligations. While 60% of eligible control participants and 62% of eligible intervention participants completed at least one aspect of the study, only 9.5% of eligible controls and 5% of eligible intervention participants completed every element of the study including all three tests. Participants who completed all aspects of the modules, tests, and the survey may have found the curriculum more valuable and/or had greater interest in movement disorders and therefore may have skewed the results positively.

If these modules were to be implemented in the future as a core component of a residency curriculum, we would recommend that careful attention is paid to accountability and engagement, even if that accountability simply came in the form of resident statements of completion where values of professionalism would hopefully trigger veracity. Our experience suggests that if this type of resource is assigned as a voluntary learning activity without accountability, a substantial portion of eligible trainees will not complete all aspects. Some programs may choose to have trainees decide individually what elements they will complete; the modules cover different topics and trainees may find value by focusing on their areas of weakness or interest. Program directors must be cognizant of time constraints for busy residents; while each of the modules can be completed within 30 minutes or less, the median time to complete the entire curriculum was 2.9 hours. In our personal experience, it may be possible to reduce this burden by including the curriculum as part of a protected didactic time frame (such as resident orientation) or asking residents to complete it during times of relatively light and relevant clinical duties such as clinic or outpatient subspecialty blocks. One way to further reduce the burden is to reduce the number of mandatory in-person didactic learning activities, especially those that cover similar educational territory.

A model similar to the flipped classroom, in which at-home pre-learning occurs before in-person learning, may be a particularly useful way to integrate new CBTMs into residency programs [5]; such an approach has been used successfully in EEG education in neurology residency [16,17]. The fact that postgraduate training level did not confer greater movement disorders knowledge on the pre-tests supports the notion that there is a relative paucity of movement disorders learning that takes place during neurology residency, consistent with the findings from semi-structured interviews with residents and fellows in our pilot study [10]. Underrepresentation of outpatient subspecialties and neurophysiology in residency training has been a consistent concern in the field [1,2]. The online, brief module-based method of teaching residents may be well suited for other outpatient subspecialties and neurophysiology training. Ideally, the pre-work (module-based curriculum) should be temporally correlated with the in-person learning experience (didactic or clinical) so that application can be immediate. This timeline would free faculty involved in the in-person learning experience to focus on more advanced concepts, capitalizing on what can be limited subspecialty exposure.

In conclusion, educational tools that allow for asynchronous learning, based on real patient videos, can allow for a standardized foundation of patient exposure in sub-specialty areas in which there is inconsistent exposure during residency. The addition of an online interactive curriculum to the existing neurology residency curriculum improved residents’ medical knowledge, and was well received by residents who found it engaging and took advantage of its inherent flexibility. Participation rates in the curriculum were highly variable between institutions, and careful attention should be paid to methods of implementation in order to maximize engagement.

## Additional File

The additional file for this article can be found as follows:

10.5334/tohm.654.s1 Supplementary Materials. Participant Survey and Modules Tests A, B, and C.
