# Supplementary material for: Multi-Residency Implementation of an Online Movement Disorders Curriculum Based on Real Patient Videos
Source: Tremor Other Hyperkinet Mov (N Y). 2021 Sep 22;11:38. doi: 10.5334/tohm.654 (PMC8462470; doi:10.5334/tohm.654)
Supplement: Supplementary Materials. — Participant Survey and Modules Tests A, B, and C. [file tohm-11-1-654-s1.pdf]

Supplementary Materials:

Participant Survey

1) What is your level of training?
    a. PGY-2
    b. PGY-3
    c. PGY-4

2) What fellowship (if any) do you plan to complete after residency?
    a. Stroke
    b. Neuro ICU
    c. Movement Disorders
    d. Neuromuscular
    e. Neurophysiology
    f. Neuro-oncology
    g. Neuroimmunology
    h. Epilepsy
    i. Headache
    j. None
    k. I don't know
    l. Other:

3) How many modules did you complete?

4) If you did not complete all modules, why not?
    i. They were too time consuming
    ii. There were too many of them
    iii. I already felt comfortable with the information
    iv. Learning about movement disorders is not a priority for me
    v. Other (please specify):
    vi. Not applicable; I completed all modules

5) Please rate the technical quality of the modules

	**Poor**	**Fair**	**Good**	**Very Good**	**Excellent**
Video Image Quality					
Video Sound Quality					
Ease to access/download					
Table of contents					
Text					
Embedded					

images (other than video)					

6) Please comment on any other aspect of the technical quality of the videos.
7) What did you like about the modules?
8) What did you dislike about the modules?
9) Please answer the following questions about how the modules affected your learning experience.

	Strongly Disagree	Disagree	Neutral	Agree	Strongly Agree	Not applicable
Completing the modules was a good use of my time						
The modules were appropriate for my learning level						
The modules helped me to understand basic concepts in movement disorders						
The modules helped me to understand difficult concepts in movement disorders						
The modules allowed me to finally "get" concepts that I had heard of before, but didn't understand.						
I feel more confident about diagnosing movement disorders as a result of this curriculum						
The modules were the right duration						
There were the right number of modules						
I enjoyed completing the modules						
Modules like these should be used for other neurology subspecialties						
I would recommend the modules to a resident in another neurology training program, or to next year's residents						

10) If you answered "neutral", "disagree," or "strongly disagree" to any of the above, please explain your response.
11) Are there any additional topics that you would have liked to see covered by the modules?

12) Please provide any other comments about the modules, including specific recommendations for improvement.

Adapted from:

1. Moeller JJ, Farooque P, Leydon G, Dominguez M, Schwartz ML, Sadler RM. A video-based introductory EEG curriculum for neurology residents and other EEG learners. MedEdPORTAL Publications. 2017;13:10570. https://doi.org/10.15766/mep_2374-8265.10570
2. Schuller MC, DaRosa DA, Crandall ML. Using just-in-time teaching and peer instruction in a residency program's core curriculum: enhancing satisfaction, engagement, and retention. Acad Med. 2015 Mar;90(3):384-91. doi: 10.1097/ACM.0000000000000578.

Modules Tests A, B, and C

Test A

1) A 45-year-old woman presents with jerky spasms of her trunk. She reports that they are the worst when she is lying down at night, but they also occur while standing. She is fully awake during the movements. The spasms cause her to flex her trunk and hips. On examination, you can induce the movement by tapping abruptly on her abdomen. What is the most likely diagnosis?
    a. Truncal dystonia
    b. Tic disorder
    c. Propriospinal myoclonus
    d. Corticobasal degeneration
    e. Conversion disorder
    •
2) What words would you use to describe chorea?
    a. arrhythmic, stereotyped, jerky
    b. arrhythmic, non-stereotyped, fluid
    c. rhythmic, stereotyped, oscillatory
    d. arrhythmic, non-stereotyped, jerky
    e. arrhythmic, stereotyped, sustained
    •
3) A 43-year-old woman comes to your office complaining of head tremor. She also feels pain in her neck and she has noticed that her head tends to twist to the left, most noticeably when she is driving or watching TV. On examination, she has hypertrophy of the left sternocleidomastoid muscle, right torticollis, left laterocollis, and a jerky no-no head tremor. Which of the following is the most appropriate initial treatment?
    a. Carbamazepine
    b. Botulinum toxin injections
    c. Baclofen
    d. Diazepam
    e. Propranolol
    •
4) When evaluating hemifacial spasm, the following test is the most likely to reveal the underlying etiology:

a. Electroencephalography (EEG)
b. Lumbar puncture
c. MRI and MRA of the head
d. Nerve conduction study (NCS)
e. Lyme titer
•

5) How do dystonia and tremor differ from one another?
a. Dystonia is co-contraction of agonist and antagonist muscles, while tremor oscillates between contractions of agonist and antagonist muscles
b. Dystonia occurs at rest, while tremor occurs with action
c. Dystonia does not resolve with sleep, while tremor resolves with sleep
d. Dystonia is treated with oral medications, while tremor is treated with botulinum toxin injections
e. Dystonia only occurs in one part of the body, while tremor can occur in many parts of the body
•

6) A 50-year-old man comes to your office for tremor. His hands shake when he is drinking from a cup or writing. He spills liquids and his writing is becoming less and less legible. This has been going on for about 10 years. He does not have rest tremor, rigidity, or bradykinesia on examination. What are the two first-line treatments for this condition?
a. Carbidopa/levodopa and topiramate
b. Tetrabenazine and gabapentin
c. Gabapentin and propranolol
d. Propranolol and primidone
e. Topiramate and primidone
•

7) A 47-year-old man comes to your office complaining of right hand tremor and poor dexterity. On examination, he has a rest tremor in the right hand, and demonstrates a decrement of speed and amplitude during finger and toe taps on the right. He has reduced right arm swing. You diagnose him with Parkinson's Disease. He is right handed and is bothered by his symptoms enough to want to start a medication. Which of the following medications is most likely to induce dyskinesias over the course of this patient's disease?
a. Rasagiline
b. Amantadine
c. Carbidopa/levodopa
d. Entacapone
e. Ropinirole
•

8) A 65-year-old man comes to the office with complaint of falls. He reports that over the past year, he feels off balance, and tends to fall when he turns around or goes up an incline. He feels that he is generally slower. It takes a long time for him to get dressed in the morning. His wife has insisted that he stop driving because he is having trouble processing everything that he has to do. On exam, what would make you most concerned for progressive supranuclear palsy as opposed to idiopathic Parkinson's disease?
a. Orthostatic blood pressures
b. Positive pull test (caught by examiner)
c. Hypophonia

d. En bloc turns
e. Dystonic foot posturing

9) A 14-year-old boy presents to your clinic reporting frequent blinking, grunting, and grimacing that started around the age of 6 years old, but has been getting worse. The movements and vocalizations are causing him social anxiety in school. Given the most likely diagnosis, for what comorbidity is this patient at greatest risk?
   a. Obsessive compulsive disorder
   b. Rett syndrome
   c. Syndenham's chorea
   d. Juvenile myoclonic epilepsy

10) What tremor characteristic is most suggestive of functional tremor?
   a. The frequency entrains to other tasks
   b. It is present only in one position
   c. It is present only in one part of the body
   d. It came on gradually
   e. It gets worse when the patient is anxious

Test B

1) A 36-year-old man comes to your office reporting that it is becoming much harder for him to play the guitar. He is in a band, and he has been making errors with his left hand. He reports cramping in his left hand when he plays, and his index finger tends to extend. He has to stop and shake out his hand and then resume playing. His band mates are getting frustrated and are threatening to replace him. He has no other symptoms. What is the most likely diagnosis?
   a. Corticobasal degeneration
   b. Stimulus-induced myoclonus
   c. Functional movement disorder
   d. Focal dystonia
   e. Asterixis
   -

2) A 56-year-old woman presents with her husband, who reports that she has been moving her head and her hands without realizing it. She has no neurological history, and no family history of neurological disease. She has a history of panic disorder, and her father committed suicide in his 60s. On examination, she has arrhythmic, fluid movements throughout her body that she cannot suppress, and delayed latency of saccades. Given the most likely diagnosis, what else is most likely to be present on your neurological exam?
   a. Hyporeflexia
   b. Alien hand syndrome
   c. Tongue protrusion impersistence
   d. Stimulus-induced myoclonus
   e. Kinetic tremor

3) What medication can be added to carbidopa/levodopa to prolong its effect?
   a. Amantadine
   b. Trihexyphenidyl
   c. Tetrabenazine

d. Ropinirole
e. Entacapone
•

4) What is a difference between tics and myoclonus?
   a. Tics are rhythmic, while myoclonus is arrhythmic
   b. Tics occur exclusively in children, while myoclonus can occur in adults or children
   c. Tics are quick and jerky, while myoclonus is a sustained contraction
   d. Tics may be temporarily suppressed, while myoclonus cannot
   e. Tics occur with action, while myoclonus occurs at rest
   •
5) A 68-year-old woman with essential tremor has tried multiple medications and still her tremor is debilitating. She spills when trying to eat or drink, and cannot write legibly. She is interested in more information about deep brain stimulation. What is the target for deep brain stimulation in essential tremor?
   a. VIM nucleus of the thalamus
   b. Globus pallidus interna
   c. Globus pallidus externa
   d. Subthalamic nucleus
   e. Putamen
   •
6) What additional neurological symptom/sign is common in a patient with hemifacial spasm?
   a. Trigeminal neuralgia
   b. Ipsilateral facial weakness
   c. Dysarthria
   d. Eye movement abnormalities
   e. Dysphagia
   •
7) A 45-year-old man presents to your botulinum toxin clinic for cervical dystonia. You ask him to sit naturally, and allow his head to move how it wants. His chin is turned to the right, his head is tilted to the left, and his head is bent slightly forward. How would you describe his head position?
   a. Right laterocollis, left torticollis, anterocollis
   b. Left laterocollis, right torticollis, anterocollis
   c. Right laterocollis, left torticollis, retrocollis
   d. Left laterocollis, right torticollis, retrocollis
   e. Right laterocollis, right torticollis, anterocollis
   •
8) A 50-year-old patient comes to your clinic for abnormal movements in his sleep. His wife has started sleeping in another bed because about twice per week he yells out and swings his arms in the bed. This occurs about 1.5 hours after sleep onset. You are concerned about REM sleep behavior disorder. For what other disorder is this patient at increased risk?
   a. Huntington’s disease
   b. Corticobasal degeneration
   c. Parkinson’s disease
   d. Essential tremor
   e. Hemifacial spasm
   •

9) Which of the following is an example of bradykinesia?
    a. Cogwheel rigidity
    b. Vertical gaze restriction
    c. Graphesthesia
    d. Micrographia
    e. Retropulsion
    -

10) A 68-year-old woman presents to your clinic for tremor. She reports that her tremor interferes with her ability to talk on the telephone, because her right hand shakes when she is holding the phone to her ear. On examination, she has a tremor in her right hand when her hands are rested in her lap, and also has a tremor in the right hand about 5-10 seconds after lifting her hands in front of her. She has no tremor when writing or pouring liquids. What is the most likely diagnosis?
    a. Essential tremor
    b. Parkinson’s disease
    c. Enhanced physiological tremor
    d. Dystonic tremor
    e. Psychogenic tremor

Test C

1) Which of the following is the best description of dystonia?
    a. Alternating contraction of agonist and antagonist muscles
    b. Pathological muscle relaxation with compensatory contraction
    c. Brief muscle contraction
    d. Stereotyped series of semi-voluntary movements
    e. Co-contraction of agonist and antagonist muscles
    -

2) A 35-year-old woman presents with facial twitching. She reports that for the last few months she has noticed that her right face will twitch uncontrollably, resulting in right eye closure. This interferes with her ability to read and drive. On examination, she has arrhythmic twitching of the right face that worsens with facial activation. Given the most likely diagnosis, what is the most appropriate initial treatment?
    a. Carbamazepine
    b. Levetiracetam
    c. Clonazepam
    d. Botulinum toxin injections
    e. Cognitive behavioral therapy
    -

3) A 75-year-old man with a history of deep venous thrombosis on warfarin presents for tremor. He has a tremor in both hands that interferes with his ability to write and drink from a cup. He is now using lids and straws, and his wife fills out checks for him. On examination, his blood pressure is 130/85, pulse 82. He has a postural and kinetic tremor in both hands, and a slight no-no head tremor. What is the most appropriate initial therapy for him?
    a. Primidone
    b. Propranolol
    c. Botulinum toxin injections
    d. Topiramate

e. Gabapentin

4) A 60-year-old woman presents to your clinic reporting imbalance. She feels off balance, and has fallen a number of times, mostly backwards while going up a step or a hill. On examination, she has rigidity in her neck more than her extremities, and a decrement of speed and amplitude of fine finger movements on the right more than the left. On primary gaze, her eyes move horizontally to one side slightly off center, pause, and then move horizontally to the other side. On pull test, she is caught by the examiner without taking a step. Given the most likely diagnosis, what would you expect to see on an MRI of her brain?
   a. Caudate atrophy
   b. Cerebellar atrophy
   c. Midbrain atrophy
   d. Unilateral cortical atrophy
   e. T2 hyperintensities in the pons
   -

5) A 68-year-old man with a 10 year history of Parkinson's disease presents for follow up. He is currently taking carbidopa/levodopa 25/250mg six times per day, every 3 hours, and entacapone 200mg with each dose. The medication only lasts for 2 hours and then he gets so stiff that he can hardly walk. When the medication is working, he has writhing movements of his head, arms, and legs, but they don't bother him much. At night, he sometimes sees animals in the corner of his room, but he knows they're not real. What is the most appropriate next step in the management of this patient?
   a. Increase carbidopa/levodopa to seven times per day
   b. Add ropinirole to increase on time
   c. Add amantadine for dyskinesias
   d. Refer for consideration of deep brain stimulation
   e. Add quetiapine for hallucinations
   -

6) When assessing for bradykinesia with finger tapping, what should you look for?
   a. A steady slowness of the movement
   b. A decrement of speed over the course of the movement
   c. Initial slowness then improvement over the course of the movement
   d. Inaccuracy of targeting the index finger onto the thumb
   e. Abnormal posturing of the fingers during the task
   -

7) A 52-year-old woman presents to your clinic for tremor. She reports that she has had tremor in both of her hands for about 5 years. What aspect of her history would help you to diagnose essential tremor rather than Parkinson's disease?
   a. She has a history of restless legs syndrome
   b. The tremor is worse on the right than the left
   c. She has a history of REM sleep behavior disorder
   d. She also has a chin tremor
   e. The tremor occurs mostly when she is using her hands
   -

8) A patient presents to your clinic complaining of head tremor and abnormal head position. On examination, she has a right head tilt and a head tremor. What aspect of the history or examination would help you to diagnose a functional origin to her symptoms?

a. The head posture improves when she lightly touches her chin
b. The tremor is jerky and irregular
c. There is limitation of left head tilt both actively and passively
d. The head posture resolves when she is tying her shoes
e. The tremor comes and goes
-

9) Other than Parkinson's Disease, name two alpha-synucleinopathies.
   a. Lewy body dementia and multiple system atrophy
   b. Multiple system atrophy and progressive supranuclear palsy
   c. Corticobasal degeneration and frontotemporal dementia
   d. Progressive supranuclear palsy and Lewy body dementia
   -

10) What is the difference between action and kinetic tremor?
   a. Action tremor is a type of kinetic tremor
   b. Kinetic tremor is a type of action tremor
   c. Action tremor occurs when using your hands to complete a task, while kinetic tremor occurs when the hands are held in a fixed posture
   d. Action tremor occurs when moving towards a target, while kinetic tremor occurs when using your hands to complete a task
   e. Action tremor is the same as kinetic tremor
